# Accurately summarizing an outbreak using epidemiological models takes time

**DOI:** 10.1098/rsos.230634

**Published:** 2023-09-27

**Authors:** B. K. M. Case, Jean-Gabriel Young, Laurent Hébert-Dufresne

**Affiliations:** ^1^ Vermont Complex Systems Center, University of Vermont, Burlington, VT 05405, USA; ^2^ Department of Computer Science, University of Vermont, Burlington, VT 05405, USA; ^3^ Department of Mathematics and Statistics, University of Vermont, Burlington, VT 05405, USA

**Keywords:** practical identifiability, epidemiological modelling, Bayesian statistics

## Abstract

Recent outbreaks of Mpox and Ebola, and worrying waves of COVID-19, influenza and respiratory syncytial virus, have all led to a sharp increase in the use of epidemiological models to estimate key epidemiological parameters. The feasibility of this estimation task is known as the practical identifiability (PI) problem. Here, we investigate the PI of eight commonly reported statistics of the classic susceptible–infectious–recovered model using a new measure that shows how much a researcher can expect to learn in a model-based Bayesian analysis of prevalence data. Our findings show that the basic reproductive number and final outbreak size are often poorly identified, with learning exceeding that of individual model parameters only in the early stages of an outbreak. The peak intensity, peak timing and initial growth rate are better identified, being in expectation over 20 times more probable having seen the data by the time the underlying outbreak peaks. We then test PI for a variety of true parameter combinations and find that PI is especially problematic in slow-growing or less-severe outbreaks. These results add to the growing body of literature questioning the reliability of inferences from epidemiological models when limited data are available.

## 1. Introduction

Incredible efforts have been made in recent years to apply epidemiological models to the empirical data borne out of the COVID-19 pandemic. The LitCovid aggregator currently contains over 3000 papers on ‘epidemic forecasting’ and ‘modelling and estimating’ trends of COVID-19 spread [1]. We are seeing similar waves of models and forecasts for recent outbreaks of Mpox, Ebola, influenza and respiratory syncytial virus. However, the enormous variability in model predictions, even among works using the same model and similar data, erodes confidence when interpreting these efforts for policy decisions [2]. It is clear that uncertainty remains about what we can expect to learn from these models, and when.

Disease models tackle the difficult challenge of describing complex epidemic processes by relating mechanistic processes to population-level observations such as daily reported cases. Identifying combinations of parameters that plausibly replicate observed data can help summarize the epidemic dynamics. Common statistics include the basic reproductive number, the average number of new cases someone will cause in an entirely susceptible population, and the outbreak size, the fraction of the population who will eventually have had the disease. Because these indicators are the product of interacting social and biological phenomena, they are never available through direct observation. Fitting epidemiological models to data is one of the best options for estimating these important quantities [3].

The classic susceptible–infectious–recovered (SIR) model accounts for a minimal number of critical mechanisms of disease spread. Infectious individuals infect susceptible individuals at a rate *β* and recover at a rate *α*. These mechanisms can be tracked through time by a set of ordinary differential equations:

$$\frac{d}{dt}S=-\beta SI,\;\frac{d}{dt}I=\beta SI-\alpha I\;\mathrm{and}\;\frac{d}{dt}R=\alpha I.$$

It is common to consider *S*, *I* and *R* as a fraction of the population in a given state such that *S* + *I* + *R* = 1 at all times. The initial state of the population might not be known—especially the susceptible pool *S*_0_ ≡ *S*(*t* = 0). Focusing on the second equation, we can see that the epidemic will grow exponentially at a rate *βS*_0_ − *α* for initial small values of *I*, resulting in near-exchangeability of the parameters and causing large uncertainty in individual parameter values early on [4,5]. Conversely, when *I* becomes small after the peak, the infectious population eventually decays exponentially at a rate *α*. These observations make clear that data regarding *I* will provide information about different parameters, or combinations thereof, at different points of an outbreak. In general, the amount of information that can be learned about a given quantity will depend on the structure of the model equations, the timing of observations and the level of noise in the data [6].

Despite the model’s simplicity, several authors have cautioned that the reliability of inferences drawn from the SIR model is questionable when based on prevalence data alone [7]. Due to the structural nature of the SIR equations, these issues are particularly acute during the early stage of an outbreak, when inferences are critical for informing timely public health response [5,8]. Without careful incorporation of additional data, these reliability problems can only grow with additional complexity in the model equations or observational structure [2,9]. In order to draw meaningful conclusions, researchers are forced to rely on data from one or more epidemic waves [10], or make strong and potentially controversial assumptions about parameters governing disease spread [11]. A more general understanding of how properties of epidemiological models affect uncertainty in commonly reported summary statistics would help researchers quantify how much they can expect to learn in empirical studies and establish sufficient criteria for reproducibility. Therefore, the goal of this report is to provide a comprehensive baseline for the reliability of estimates for a number of commonly reported statistics, with emphasis on the time necessary to predict these statistics in an emerging epidemic accurately and to illuminate the structural interactions between data, model dynamics and summary statistics.

This question of whether quantities estimated from data are reliable, e.g. compatible with some hypothetical true parameters $\theta^{*}=(\alpha^{*},\beta^{*},S_{0}^{*})$ which generated the data, is termed the practical identifiability (PI) problem and has traditionally been studied using the variance–covariance matrix of an estimator for $\theta^{*}$ [12]. However, such second-order approaches underestimate uncertainty in limited data settings, where the distribution of plausible parameters may be skewed [13,14]. Here we propose a new measure that allows us to efficiently and directly measure our ability to learn various epidemiological quantities at all stages of an epidemic. If u=f (θ) is an unknown variable to be estimated, our Bayesian interpretation of the identifiability of *u* is the expected logarithm of the ratio between posterior and prior probabilities, evaluated at $u^{*}=f\;(\theta^{*})$:

1.1 $$\delta_{u}(\theta^{*})=E_{y|\theta^{*}}[\log P(u^{*}|y)-\log P(u^{*})],$$

where $y|\theta^{*}$ are noisy observations of the underlying outbreak, e.g. daily case counts, and where the expectation is taken over realizations of the observation process. Since shrinkage in the posterior distribution is facilitated through the global behaviour of the model likelihood, (1.1) is able to capture uncertainty arising from complex model fits, such as bimodality in the likelihood surface. As with traditional approaches to PI, *δ*_*u*_ is a local measure of information gain, in the sense that changing the true dynamics $\theta^{*}$ will, in general, give different answers [15]. This allows the effect of particular values of $\theta^{*}$ to be studied. Note that the metric does not require computationally expensive Bayesian inference methods to compute—a simple Monte Carlo procedure for estimating (1.1) is provided in appendix A.

## 2. Results

**Figure 1.**
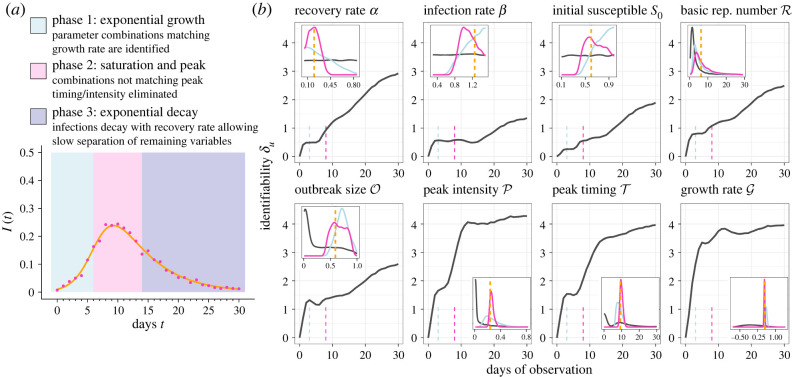
Practical identifiability of epidemiological summary statistics over time. (*a*) Unknown deterministic SIR process based on true parameters $\theta^{*}$ (orange line), and single realization of observed data $y∼P(y|\theta^{*})$ (pink dots). (*b*) Main panels show PI according to *δ*_*u*_ over an increasing observation window assuming daily observations. Insets give an example of how *δ*_*u*_ is interpreted, showing *P*(*u*|**y**) and *P*(*u*) for the single realization of **y** from (*a*), observed up to *T* = 3 (blue) and *T* = 8 (pink). The dashed orange line is the true value to be estimated. True parameters are α* = 0.2, β* = 1.25 and $S_{0}^{*}=0.6$, with *I*_0_ = 0.01 assumed known. Prior beliefs are *α* ∼ *U*(0.05, 0.85), *β* ∼ *U*(0.3, 1.5), *S*_0_ ∼ *U*(0.1, 0.99).

Figure 1 shows the PI of the SIR model parameters, as well as five summary variables which are commonly calculated in terms of θ (see table 1 for mathematical definitions), for a typical parametrization $\theta^{*}$ of the model. Infectious individuals are assumed to be independently tested at a fixed rate *η* at daily timepoints, giving a likelihood $y_{t}∼\mathrm{Poisson}(\eta I(t;\theta^{*}))$. We assumed *η* = 1000 is known throughout, which leads to limited observational noise to better study PI inherent to the SIR equations. *δ*_*u*_ is computed daily for the eight variables, up to a maximum of 30 days of observation.

**Table 1.** Definitions of epidemiological summary statistics.

name	symbol	formula
reproductive number	R	*β*/*α*
outbreak size	O	$1-R(0)-S_{0}\exp{\left(-RO\right)}^{a}$
peak intensity	P	$I_{0}+S_{0}+[1-\log(S_{0}/R)]/R$
peak timing	T	unknown
growth rate	G	*βS*_0_ − *α*

^a^Implicit equation.

The rate of learning for all variables is uneven over time, with each reaching plateaus of varying length before the peak. The infection rate *β* is the worst identified. Gaining information on *α* appears easier than *β* and *S*_0_ and even exceeds learning for R and O after around *T* = 20 days of observation. PI of the peak intensity, peak timing and growth rate increase more rapidly at first, with learning for growth rate happening particularly fast.

**Figure 2.**
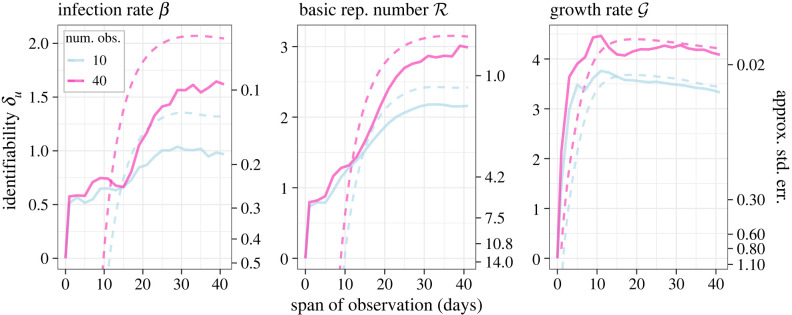
Practical identifiability of several variables as a function of testing frequency. Observations are evenly distributed over the interval [0, *T*] for increasing days of observation *T*. Solid lines are PI calculated using Monte Carlo, while dashed lines indicate the approximation given by (B 3). The approximation also gives an asymptotic relationship between *δ*_*u*_ and a lower bound on the standard error, indicated with secondary axes. Priors and true parameters are the same as in figure 1.

These findings illustrate the difficulty of learning key quantities early in an epidemic, under real-time conditions where the number of observations increases as the outbreak goes on. However, the question remains as to what extent a lack of early learning may be attributed simply to a smaller sample size. Therefore, we next examined the PI of several variables over an increasing observation window, but with the number of evenly distributed observations kept constant. Figure 2 shows that identifiability of *β* and R is lowest when observations are concentrated prior to the peak, confirming that the limits of early learning are indeed a structural property of the SIR equations that cannot be overcome by allocating additional tests early on. Further, increasing the frequency of testing from 10 observations to 40 did little to increase PI during this period, but increased PI considerably for wider observation windows. Figure 2 also shows the functional relationship between the asymptotic limit of *δ*_*u*_ and the usual standard error for *u*, as given by (B 3), which can serve as an alternative interpretation of *δ*_*u*_ when there are sufficient data.

**Figure 3.**
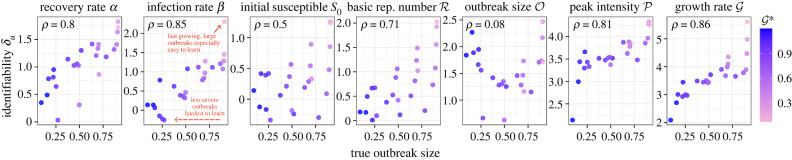
Change in PI *δ*_*u*_ when the true parameters $\theta^{*}$ are varied. *δ*_*u*_ is calculated using daily observations up to the first day after the true (unobserved) outbreak has peaked. Each dot corresponds to a combination of true parameters with β* = 0.3, 0.5, …, 1.5 and $S_{0}^{*}=0.1,0.3,\ldots ,0.9$ while α* = 0.2 is fixed. Pearson correlation between *δ*_*u*_ and true outbreak size is given in the corners of each panel. Priors are the same as in figure 1.

To test the sensitivity of these findings to $\theta^{*}$, we then computed *δ*_*u*_ over a grid of values for β* and $S_{0}^{*}$ (figure 3). Since slower-growing outbreaks will naturally contain less information per day [7], information gain was calculated using observations up until the first day after the epidemic peak. To investigate the factors of a true outbreak most associated with learning, for each true value of the eight variables considered, the correlation between *δ*_*u*_ for each variable and the true value was computed. The outbreak size of the true epidemic was the most correlated with learning, followed by true growth rate, illustrating that less-severe outbreaks are harder to learn.

## 3. Discussion

The analysis presented here makes it clear that some epidemiological variables are easier to estimate through model dynamics than others, and emphasizes that most epidemiological summary statistics should be interpreted with caution when data are limited. Taken together, the rate of learning for all the variables suggests that learning takes place in three general phases. In Phase 1, plausible parameter combinations quickly concentrate along the surface $\left\{\theta \;:\;\beta S_{0}-\alpha =G^{*}\right\}$, as infections increase exponentially with the initial growth rate. This explains the sharp but modest gain in the information of all variables except for G during this phase. In Phase 2, infections begin to saturate and parameter combinations matching the true peak intensity and timing become more plausible. However, for *β* especially, saturating case counts do little to further restrict the plausible parameter surface from Phase 1. Finally, Phase 3 is characterized by gradual information gain for the remaining variables. Since infections are slowly declining with *α* during this phase, this growth is explained by α* gradually being identified, which propagates to allow some remaining combinations on the plausible surface to be eliminated.

Parameters describing the mechanisms of the model—*β*, *α* and *S*_0_—take a particularly long time to learn on account of quickly reaching a plateau at low values of *δ*_*u*_. As a result, the SIR model is more effective at forecasting short-term statistics of the dynamics, such as peak timing and intensity, than it is at estimating mechanisms. This result shows how difficult it is to estimate parameters from early data in the hope of forecasting the impacts of mechanistic interventions such as reducing *β* with preventive measures or increasing *α* with treatment [16]. Importantly, even though a lack of identifiability implies a wide range of parameters lead to similar infectious dynamics early on, these plausible dynamics will still respond differently to interventions targeting specific mechanisms [17]. Thus, low PI simply means that an intervention’s impact is difficult to forecast ahead of time.

Learning was nearly as difficult for the statistics R and O as for the individual model parameters, despite the fact that optimistically, these transformations would combine the information of each parameter they depend on. The failure of these statistics to resolve closely exchangeable parameter combinations limits their reliability for succinctly describing an epidemic. By contrast, the initial growth rate resolves such combinations to give rapid shrinkage to the correct value, despite encoding similar information to R about disease dynamics [18]. This suggests growth rates are a more reliable ‘first look’ at an outbreak when using prevalence data under the SIR model.

When varying the true values $\theta^{*}$, see figure 3, we find that less-severe outbreaks are generally harder to learn, despite having more daily observations available before their peak. The initial susceptible population *S*_0_ appears the most poorly identified across values of $\theta^{*}$ by the peak, and the expected posterior shrinkage is even slightly negative for 25% of the tested values. An interesting implication for control measures is that the more we reduce the severity of true infection dynamics, the harder it will be to accurately estimate the impacts of interventions. Further, the mode of intervention matters: variability along the *y*-axis in figure 3 for similar values of $O^{*}$ shows lowering $S_{0}^{*}$ impacts learning differently than a reduction in β*.

Previous investigations into the PI of the SIR model have mainly focused on the PI of *α* and *β* under the simplified model where *S*_0_ ≈ 1 is known. These works generally agree that PI of both *α* and *β* is limited during Phase 1 [4,5], but that the majority of information available has been learned by the time the disease has peaked [9,19,20]. Most comparably to the observational design in figure 1, Capaldi *et al.* [7] considered the asymptotic variance of $\hat{\beta }$ and $\hat{\alpha }$ over an increasing timespan, and found the variance of both estimators decreased rapidly and smoothly just before and after the peak, respectively [7]. In contrast, the uneven rate of learning of these parameters in figure 1 paints a more nuanced and pessimistic picture of PI when exact likelihoods and prior context are taken into account. This finding supports the idea that previous PI results based on approximation theory underestimate uncertainty, particularly during the early stages of an outbreak when the likelihood surface is highly nonlinear [13,21].

In this work, we have proposed a novel means of assessing PI which measures the expected posterior gain in density at the true value u*. While comparing densities at a specific value may seem to ignore uncertainty in the posterior as a whole, we argue that *δ*_*u*_ is better interpreted as a measure of *shrinkage* rather than density, by marginalizing the global curvature of the likelihood onto a single dimension for *u*. If the projected span of high likelihood values is more narrow than the support of *P*(*u*), shrinkage will occur and *δ*_*u*_ becomes positive. In this sense, (1.1) might be viewed as a quantitative alternative to the popular profile likelihood method, in which potential plateaus in the likelihood surface are projected to the space of some parameter *θ*_*i*_ and examined graphically [22]. Additionally, as shown in appendix B, *δ*_*u*_ may be interpreted in terms of standard measurements of uncertainty—in the limit of large data and under certain conditions, *δ*_*u*_ converges to a density form of the usual standard error of the maximum likelihood estimator, penalized by the prior weight. Therefore, while our measure was specifically designed to give a more accurate picture of uncertainty in limited data regimes, it also has asymptotic behaviour similar to the coefficient of variation for *u*.

The Bayesian nature of our method of assessing PI means that estimates of model parameters and any variables which depend on them are sensitive to prior beliefs. In this report, our choice of uniform priors represents modest assumptions about an emerging pathogen: *a priori*, just over 50% of scenarios result in an outbreak (i.e. have *βS*_0_/*α* > 1), and outbreaks range from modest to highly severe (70% of individuals infected at peak). However, for many pathogens, more informative prior information is frequently available, for example on the recovery rate of a disease [23]. Relative to more realistic prior settings, this may mean *α* is more difficult to gain information about than *β* and *S*_0_.

While we have considered only noisy observation of the current infectious population, real data may also come in the form of daily new infections or cumulative case counts, and may suffer from lags in reporting or preferential sampling [24,25]. Learning epidemiological variables from such data will have their own distinct challenges [9]. PI of the SIR model should also be assessed with hierarchical models incorporating data from multiple sources, such as hospitalizations and isolated clinical experiments [26]. Yet, our work shows that even in its simplest form, learning parameters and statistics of SIR dynamics takes time, limiting which inferences, forecasts and control policies can be made from early epidemic data.

## Figures and Tables

**Figure 4.**
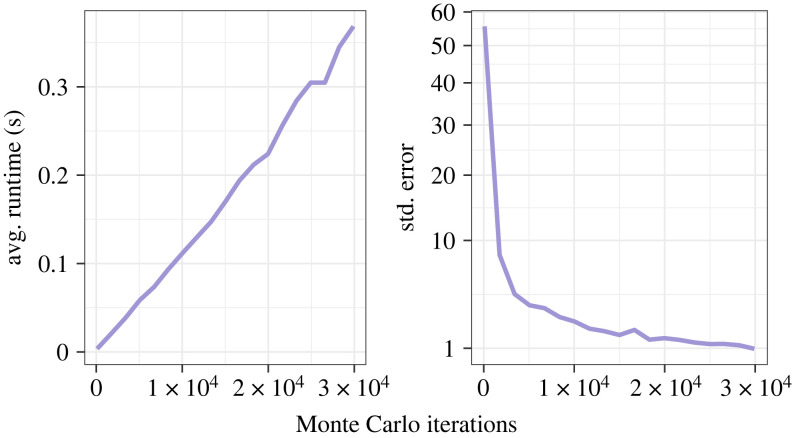
Speed and accuracy of approximating the log marginal likelihood log *P*(**y**) using *M* Monte Carlo simulations. The results are averaged over 100 repetitions of the sampling process for each *M*.

**Figure 5.**
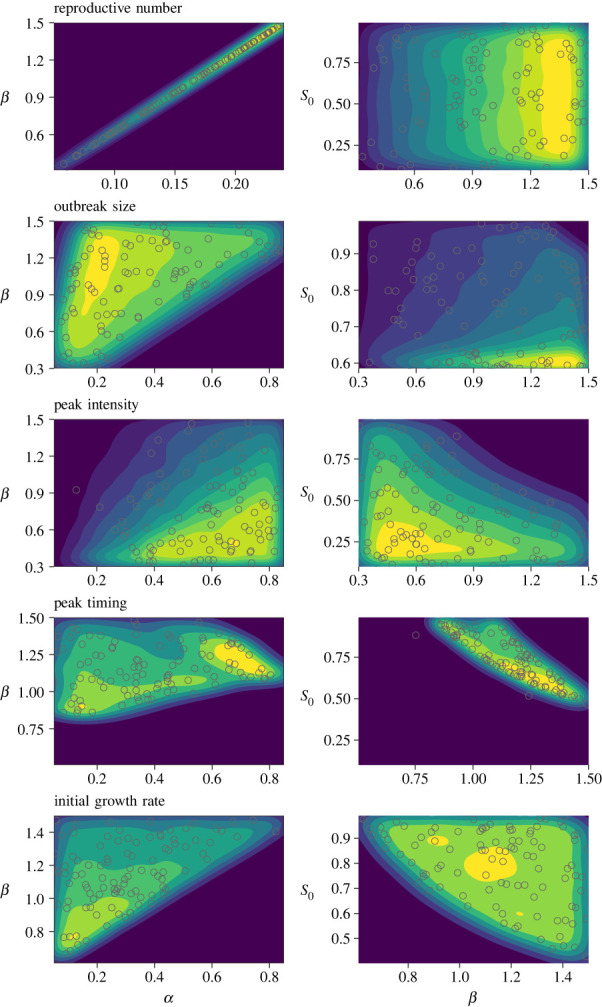
Density of 60 000 samples from *P*(*α*, *β*| u*), and *P*(*β*, *S*_0_| u*) given different summary transformations. True values and priors are the same as in the main text.

## Data Availability

Materials necessary to reproduce this analysis are available on GitHub and have been archived on Zenodo [27]. A Julia package MarginalDivergence.jl has been developed for efficient computation of our PI measure, including an interface for easy implementation of user-provided models. The package is currently unregistered.

## Appendix A. Supplemental methods

**A.1. Likelihood-based estimation of dynamical systems**

While the methods considered here can be applied to any statistical process for which a likelihood exists, we are interested in processes of the form

A 1 $$y_{i}∼g(x(t_{i}),\sigma )$$

and

A 2 $$\dot{x}=h(x(t),\tau )$$

where **y** = (*y*_1_, …, *y*_*n*_) are observations at discrete timepoints *t*_1_, …, *t*_*n*_, and σ, τ are parameters that are assumed known or are to be estimated. We refer to *h* as the *latent process* and *g* as the *observation process*. We are interested in our ability to estimate a set of unknown parameters $\theta^{*}\subseteq (\sigma^{*},\tau^{*},x{\left(0\right)}^{*})$.

Given θ, equations (A 1)–(A 2) form a probability distribution P(y∣θ) called the *likelihood*. In the frequentist paradigm, an estimator for $\theta^{*}$ can be obtained by maximizing P(y∣θ):

$${\hat{\theta }}_{\mathrm{MLE}}={\text{argmax}}_{\theta }P(y|\theta ).$$

A popular way to assess issues of PI is through the variance–covariance matrix of ${\hat{\theta }}_{\mathrm{MLE}}$, which can show marginal uncertainty in individual parameter estimators and correlations between pairs of estimators. The Cramer–Rao bound implies that in the limit of decreasing observation uncertainty (i.e. as the amount or precision of data increases), the variance of an unbiased estimator converges, given certain regularity conditions, to the inverse of the Fisher information matrix $I(\theta^{*})$, where

A 3 $${\left[I(\theta )\right]}_{ij}=-E_{y|\theta }\left[\frac{\partial^{2}}{\partial \theta_{i}\partial \theta_{j}}\log P(y|\theta )\right].$$

This bound can underestimate variance when measurement noise is not infinitesimal [14,21], leading some to question its applicability even for simple nonlinear models [13,28]. An alternative is to estimate the distribution of ${\hat{\;\theta }}_{\mathrm{MLE}}$ using Monte Carlo simulation, by sampling possible datasets $y^{\left(1\right)},y^{\left(2\right)},\ldots$ from $P(y|\theta^{*})$ and finding the maximum of each likelihood $P(y^{\left(j\right)}|\theta )$ using an optimization algorithm such as gradient descent. The resulting samples ${\hat{\theta }}_{\mathrm{MLE}}^{\left(j\right)}$ can then be inspected graphically or used to estimate the covariance matrix. This method has the convenience of also working with estimates of transformations of the model parameters, without the need for further approximation [19,29].

**A.2. Proposed method of assessing practical identifiability**

While using Monte Carlo estimation of $\mathrm{Var}({\hat{\theta }}_{\mathrm{MLE}})$ to assess PI can alleviate the underestimation issues when using the information matrix, the use of optimization to obtain a sample of the estimator can lead to dependence on initial conditions or other hyperparameters of the optimization method used [12]. Again, the inaccuracy of this method will be most acute when the likelihood surface is flat or multi-modal, such as when limited data are available.

Rather than relying on optimization, we instead take a sampling-based Bayesian perspective. From the main text, we have for a variable of interest $u=f\;(\theta ),\delta_{u}(\theta^{*})=E_{y|\theta^{*}}[\log P(u^{*}|y)]-\log P(u^{*})$, which gives the average amount, over possible future outbreaks $P(y|\theta^{*})$, a researcher can expect to learn about the true quantity *u** in a Bayesian analysis. A value of *δ*_*u*_ = *c* corresponds roughly to an expected gain in posterior probability *c* orders of magnitude greater than the prior.

Equation (1.1) can be rewritten by applying Bayes’ rule, *P*(*u**|**y**)/*P*(*u**) = *P*(**y**| *u**)/*P*(**y**), where the margin *P*(**y**| *u**) equals $\int P(y|\theta )P(\theta |u^{*})d\theta$ and $P(\theta |u^{*})$ is the distribution of the epidemiological parameters compatible with a fixed variable of interest *u**—we give details below. This leads to

A 4 $$\delta_{u}(\theta^{*})=E_{y|\theta^{*}}\left[\log\frac{P(y|u^{*})}{P(y)}\right].$$

We approximate $\delta_{u}(\theta^{*})$ by generating *M* paired Monte Carlo samples from $P(\theta |u^{*})$ and P(θ), and reusing these samples to obtain *M* samples from *P*(**y**| *u**) and *P*(**y**) for each $y∼P(y|\theta^{*})$, leading to

$$\delta_{u}(\theta^{*})\approx \frac{1}{N}\sum_{i=1}^{N}[\log\sum_{\;j=1}^{M}P(y^{\left(i\right)}|{\tilde{\theta }}^{\left(j\right)})-\log\sum_{\;j=1}^{M}P(y^{\left(i\right)}|\theta^{\left(j\right)})]$$

where ${\tilde{\theta }}^{\left(j\right)}∼P(\theta |u^{*})$, $\theta^{\left(j\right)}∼P(\theta )$ and $y^{\left(i\right)}∼P(y|\theta^{*})$. *N* = 3000 and *M* = 60 000 were used for all computations in the main text.

**A.2.1. Accuracy of Monte Carlo estimation of *δ*_*u*_**

The marginal likelihood *P*(**y**) is notorious for being inefficient to estimate via Monte Carlo methods. To test our choice of *M* was large enough while still within a reasonable computational budget, we repeated calculations of log *P*(**y**) for increasing values of *M*, where a $y∼P(y|\theta^{*})$ was sampled with 60 observations (every half day). $\theta^{*}$ and P(θ) were the same as in figure 1 of the main text. We concluded that even with 60 observations, which gives a likelihood sharper than the maximum 30 observations used in the main text, a choice of *M* > 30 000 was sufficient to give a standard error less than 1, or less than 0.5% of the magnitude of log *P*(**y**). The runtime and standard errors from 100 independent computations of *P*(**y**) are shown as a function of *M* in figure 4.

**A.3. Practical identifiability for a function of model parameters**

The distribution function $P(\theta |u^{*})$ will generally not be available in closed form even when P(θ) is.

Simulating from $P(\theta |u^{*})$ can be accomplished with the following procedure: let $\theta_{i}\in \theta$ be a chosen ‘pivot’ parameter/index and define $\tilde{f}(\theta_{i}|\theta_{-i})=u$ to be a univariate function conditional on $\theta_{-i}$, where $\theta_{-i}$ indicates the *i*th element of θ has been removed. Assume $\tilde{f}$ is invertible so that ${\tilde{f}}^{-1}(u|\theta_{-i})=\theta_{i}$. Then, assuming independent priors on the elements of θ, using a change of variables and Bayes’ rule we have

A 5 $$P(\theta_{-i}|u^{*})\propto \prod_{\;j\neq i}P(\theta_{j})P(u^{*}|\theta_{-i}),$$

where

A 6 $$P(u|\theta_{-i})=\left|\frac{d}{du}{\tilde{f}}^{-1}(u|\theta_{-i})\right|P_{\theta_{i}}({\tilde{f}}^{-1}(u^{*}|\theta_{-i})).$$

Because $\tilde{f}$ is deterministic (i.e. *θ*_*i*_ can be uniquely determined given $\theta_{-i}$ and *u*), samples from $P(\theta |u^{*})$ can, therefore, be obtained by first sampling $\theta_{-i}^{\left(1\right)},\ldots ,\theta_{-i}^{\left(n\right)}$ from (A 5) using a standard simulation technique such as accept–reject sampling, and then letting $\theta_{i}^{\left(j\right)}={\tilde{f}}^{-1}(u^{*},\theta_{-i}^{\left(j\right)})$. The resulting densities for the five transformations considered here are shown in figure 5.

For example, under the transformation f (θ)=β/α= : R, we define ${\tilde{f}}^{-1}(\alpha ,S_{0},R)=\alpha R$ and obtain

A 7 $$P(\alpha ,S_{0}|R)\propto P_{\alpha }(\alpha )P_{S_{0}}(S_{0})\alpha P_{\beta }(R\alpha ).$$

So we may sample $(\alpha^{\left(1\right)},S_{0}^{\left(1\right)}),(\alpha^{\left(2\right)},S_{0}^{\left(2\right)}),\ldots$ from (A 7), then let $\beta^{\left(i\right)}=R^{*}\alpha^{\left(i\right)}$ to obtain a sample from $P(\alpha ,\beta ,S_{0}|R^{*})$.

For the final outbreak size, we define O : =R(∞)−R(0) to be the total proportion of individuals who end up in the recovered compartment due to infection. For *R*(∞), we have from Weiss [30]

A 8 $$R(\infty )=1-S_{0}\exp(-R(R(\infty )-R(0))),$$

which we may use to solve for *β* and obtain the inverse function

A 9 $$\beta =\frac{-\alpha }{O}\log\frac{1-R(0)-O}{S_{0}},$$

and the derivative

A 10 $$\frac{d\beta }{dO}=\frac{\alpha }{O}\left(\frac{1}{O}\log\frac{1-R(0)-O}{S_{0}}+\frac{1}{1-R(0)-O}\right).$$

For the peak intensity $P\;:=\max_{t}I(t)$, to obtain samples from (A 5) we may use the equation

A 11 $$P=I_{0}+S_{0}-\frac{\alpha }{\beta }\log S_{0}-\frac{\alpha }{\beta }\left(1+\log\frac{\alpha }{\beta }\right).$$

Although (A 11) yields only implicit solutions for any *θ*_*i*_, a closed-form solution for *S*_0_ given P can be found using Lambert’s *W*:

A 12 $$S_{0}=-R^{-1}W_{-1}(-B),$$

where $B=\exp(-R(P-I_{0})-1)$, and derivative

A 13 $$\frac{dS_{0}}{dP}=\frac{1}{1-B\;e^{W_{-1}(-B)}}.$$

Derivation of the necessary equations for the initial growth rate $G\;:=\beta S_{0}=\alpha$ is straightforward.

Finally, the peak timing T does not have a known closed-form solution. Though more time-consuming, we can still approximate (A 5) by using univariate constrained optimization to evaluate the unknown *f*^−1^, and adjoint methods to obtain the corresponding derivative.

## Appendix B. Asymptotic properties of practical identifiability

The above procedure for estimating *δ*_*u*_ using simple Monte Carlo becomes inefficient when the dimension of θ or **y** becomes large. In the latter case, the proposed method of PI can instead be analysed using the usual approximation theory in the limit of large data. The Bernstein–von Mises theorem gives

B1 $$P(\theta |y)\approx N({\hat{\theta }}_{\mathrm{MLE}},{\left(n_{t}I({\hat{\theta }}_{\mathrm{MLE}})\right)}^{-1}),$$

where *n*_*t*_ is the number of independent replications of the time series of observations, and N(μ,Σ) is the multivariate normal distribution with mean μ and covariance matrix Σ. Under certain regularity conditions, ${\hat{\theta }}_{\mathrm{MLE}}=\theta^{*}$.

The information matrix of the model parameters can be separated in terms of the curvature of latent and observation processes. In the case where data from a single state variable *x* are observed, $I(\theta )=J^{⊤}O(\theta )J$, where **J** is the Jacobian of *x* with respect to θ, $J_{ij}=\partial x(t_{i})/\partial \theta_{j}$, and O(θ) is the information of **y** given *x*. In the case of independent Poisson-distributed testing, $O(\theta )=\mathrm{diag}(\eta I(t_{1};\theta ),\ldots ,\eta I(t_{n};\theta ))$.

To obtain an approximation for the posterior of a transformation u=f (θ), we may again choose a pivot parameter/index *θ*_*i*_ and introduce a change of variables $v=(\theta_{1},\ldots ,\theta_{i-1},u,\theta_{i+1},\ldots ,\theta_{p})$, and define the vector-valued function $\tilde{f}$ so that $\tilde{f}(v)=\theta$. Letting **F** be the gradient of $\tilde{f}$ with respect to **v**, **F**_*kj*_ = ∂*θ*_*k*_/∂*v*_*j*_, we have

B2 $$I(v)=F^{⊤}J^{⊤}O(\theta )JF.$$

Combining (B 1) and (B 2), therefore, gives the approximation

B3 $$\delta_{u}(\theta^{*})\approx \frac{1}{2}(\log n_{t}-\log I{\left(v^{*}\right)}_{ii}^{-1}-\log(2\pi ))-\log P(u^{*}).$$

This reveals that, in the limit of sufficient data, *δ*_*u*_ is related to the local curvature of the latent and observational processes, just like traditional asymptotic approaches to PI. For individual model parameters u∈θ, **F** is the identity matrix and *δ*_*u*_ becomes the logarithm of the usual standard error of the estimator ${\hat{u}}_{\mathrm{MLE}}$, penalized by the prior log-probability of ${\hat{u}}_{\mathrm{MLE}}$. For parameter transformations, the information of I(θ) is then summarized further through the curvature induced by the transformation function *f*. Also note that penalizing by the prior density has a normalizing effect, as transformations that increase the support of *P*(*u*) will also have smaller prior densities, and therefore is analogous to using the coefficient of variation to allow comparing standard errors between variables.
